# Implementing Gaussian process modelling in predictive maintenance of mining machineries

**DOI:** 10.1177/25726668241275434

**Published:** 2024-08-30

**Authors:** Zhixuan Shao, Mustafa Kumral

**Affiliations:** 1 Mining and Materials Engineering Department, 5620 McGill University, Montréal, QC, Canada

**Keywords:** mining machine, predictive maintenance, Gaussian process, sensor-data diagnosis and prognosis, maintenance decision support

## Abstract

Mining machinery constitutes essential assets for a mining corporation. Due to economies of scale, technological innovations and stringent quality and safety requirements, the size, complexity, functionality and diversity of industrial machinery have expanded markedly over the last two decades. This growth has increased sensitivity to machine availability and reliability. Mining operations install comprehensive maintenance units tasked with inspection, repair, replacement and inventory management for the machines in use. Leveraging the proliferation of sensor technologies integrated within the machines, maintenance units obtain rich data streams synchronously disclosing machine health and performance metrics, which enables a predictive maintenance programme. This programme performs prognostic detections of anomalies and permits timely intervention to avert catastrophic breakdowns. However, such sensor-driven predictive maintenance scheme for machinery in the mining sector is limited. The present paper utilises the Gaussian process, a powerful predictive modelling technique, to show its potential in addressing this challenge. The efficacy of this approach is validated through three case studies. Each case study is equipped with sensor data and represents a typical predictive maintenance task for mining assets. The developed Gaussian process models successfully capture meaningful temporal patterns in sensor data and generate credible predictions across all three tasks: temporal prediction of sensor data degradation trends, remaining useful lifespan prediction and simultaneous monitoring and prediction of multiple machine conditions. Furthermore, the models offer uncertainty estimates to the prediction outcomes, potentially facilitating maintenance decision-making process.

## Background

Mining machines are complex systems used in the different stages of a mining operation. Effective management and functioning of these assets, empowered by continuing data-driven advancements (Anaraki and Afrapoli, 2023; Hazrathosseini and Moradi Afrapoli, 2023; Novoseltseva et al., 2023; Park and Choi, 2024; Tao et al., 2022), are critical for maintaining the required productivity and maximising operational efficiency. Mining machinery typically consists of a multitude of components that are subjected to repetitive usage, which can lead to various abnormalities and failures over time. These components, such as engines, hydraulics and mechanical parts, undergo significant stress and wear during operation in harsh mining environments. Thus, it is essential to detect and address these potential issues proactively in the early stage in order to prevent minor problems from escalating into major failures.

Traditionally, mining organisations have relied on reactive or scheduled maintenance strategies to manage the upkeep of their machinery. Unfortunately, these approaches, though widely adopted, do not align with proactive maintenance activities as previously discussed. Reactive maintenance deals with an emergent failure as it appears. Therefore, machinery can encounter sudden breakdowns and safety incidents. Scheduled maintenance, to some extent, alleviates this issue by servicing a machine respecting an interval regulated by the original equipment manufacturer or based on mechanicians’ experiences. Nevertheless, following a pre-defined service schedule can lead to unnecessary downtime and resource allocation, since it overlooks the distinctive maintenance needs of components or systems that adhere to diversified deterioration rates. In addition, this strategy is unable to foresee emerging issues before they escalate into major failures.

The increasing presence and employment of sensor technologies in mining machinery industries render maintenance in a prognostic way possible. Generating abundant data streams, the installed sensors can continuously monitor various key performance indicators of the related component or system, including temperature, vibration, noise, humidity level, lubrication level and oil pressure, thereby providing real-time insights into machinery health and performance. The harnessing of sensor-driven diagnostic data revolutionises maintenance activities as it allows for projecting maintenance activities into the future through leveraging the wealth of diagnostic sensor information. The resultant maintenance strategy is commonly referred to as ‘predictive maintenance’. Predictive maintenance performs early identification of machinery abnormalities through advanced analytics on historical and real-time data. The aim is to extract valuable signs and patterns that emerge over time as the machine undergoes prolonged operation. The signs and patterns will disclose the health dynamics of the component or the system. The maintenance team will benefit from this valuable prognosis to prioritise maintenance activities, allocate resources efficiently and mitigate risks associated with unplanned downtime and costly repairs.

Despite the great promise of predictive maintenance for the mining industry, related studies conducted over the years are limited. Carstens (2012) applied a proportional hazards model as a prognostic approach performing predictions on the remaining operational duration of haul truck engines. Dong et al. (2017) proposed a predictive maintenance system for mining ventilator equipment by integrating the Internet of Things. Zhong et al. (2018) argued a hybrid method for fault identification in coal mine shearer equipment. The method performed principal component analysis for dimension reduction, followed by a neural network empowered by Adaboost, which showed high accuracy in fault diagnosis. Taghizadeh Vahed et al. (2019) applied an enhanced K-nearest neighbour approach combined with a genetic algorithm on predictive maintenance task for the mining dragline. Robatto Simard et al. (2023) indicated the significance of predictive maintenance through interviews with practitioners working in several Canadian mines. Kazemi et al. (2023) implemented two XGBoost-based models to predict the time to failure for open-pit shovel machines. The model used the particle swarm optimisation shows its superiority in the study. Canelón et al. (2024) presented a holistic prognosis framework that provided remote maintenance assistance for mining trucks. Despite these advancements, there remains a noticeable gap in predictive maintenance implementations concerning the effective modelling of temporal relationships within sensor data and the quantification of uncertainty in prediction outcomes. Temporal relationships describe the connections and trends between data points over time. They show how past observations influence future observations, reflecting the dynamic nature of machinery operations. Grasping temporal relationships is crucial for a predictive maintenance model to understand the evolving health status of components and conduct accurate predictions about future maintenance needs. On the other hand, due to factors such as sensor noise, measurement errors and the variability of machinery behaviour, a predictive maintenance model cannot always guarantee deterministic forecast outcomes. As such, the integration of uncertainty analysis is essential for a predictive maintenance programme. By quantifying the uncertainty associated with model predictions, such analyses provide insights into the reliability and confidence levels of the forecasts. This, in turn, empowers maintenance teams to make informed decisions regarding resource allocation and scheduling.

Gaussian process is a machine-learning technique and a fundamental component of Bayesian probabilistic modelling. It offers several distinct advantages. As a kernel-based strategy, Gaussian process is proficient at identifying and modelling complex, non-linear relationships in data without pre-defined functional forms. Meanwhile, it can be adapted to diverse data behaviours based on kernel(s). Such flexibility and versatility are often lacking when using linear regression or simple time series models, e.g. autoregressive integrated moving average. Another credit lies in Gaussian process's ability to incorporate uncertainty estimates into prediction outcomes due to the probabilistic nature of the method. This feature provides insights into the confidence levels of predictions, which are often crucial for risk management and decision-making in predictive maintenance. In contrast, techniques such as decision trees and random forests typically do not offer explicit uncertainty estimates. Neural networks, including deep learning models, also do not inherently provide uncertainty estimates without additional modifications. Furthermore, hyperparameters in the Gaussian process are intuitive in showing the model's assumptions about the data, and their optimisation process is typically straightforward and interpretable. Neural networks, however, often suffer from opaque and complicated hyperparameter controls. Additionally, unlike many conventional machine-learning techniques that require large training data for robust prediction performance, the Gaussian process excels in scenarios where data collection is constrained or costly. Thanks to its Bayesian nature, this approach allows for the integration of prior knowledge and dynamic updates of predictions based on observations. Consequently, the dependability of predictions is maintained even with limited sensor data training inputs.

A broad application of the Gaussian process is observed in various disciplines and sectors. Clifton et al. (2012) showed the scenario of using Gaussian process for patient e-health monitoring empowered by wearable sensors. Based on small-scale training data, Frey and Osborne (2017) employed a Gaussian process model that manages to distinguish the susceptibility to computerisation of 702 occupations from the US labour market. Studies have been done through Gaussian process in battery health prediction (Li et al., 2020; Liu and Chen, 2019; Richardson et al., 2017; Tagade et al., 2020). Kang et al. (2017) showed a work where the Gaussian process is used in the evaluation and prediction of slope stability. Laib et al. (2018) developed Gaussian process models to forecast natural gas consumption in Algerian market. Benker et al. (2021) and Zeng et al. (2021) showed the feasibility of Gaussian process in predicting the remaining lifespan of turbofan engine. Guan et al. (2020) applies the Gaussian process in an application associated with air pollution prediction using vehicular monitors. Cui et al. (2021) applied the Gaussian process in the estimation of groundwater salinity in Australia. In the mining sector, the application of Gaussian process is highly limited. The research in Dong (2012) focuses on the monitoring and prediction of coal mine gas emission based on a Gaussian process model. Pu et al. (2020) built a Gaussian process model to predict the cemented rockfill strength in backfilling operations. Arthur et al. (2020) and Fissha et al. (2023) applied Gaussian process in the identification and forecast ground vibrations induced by blasting activities in mining. In the context of mining machinery predictive maintenance, the research remains scarce.

The main objective of this paper is to demonstrate the Gaussian process as a solid predictive maintenance strategy for mining machineries. The research is built on three case studies related to three representative predictive maintenance scenarios: (1) the machinery degradation trend analysis and prediction, (2) the estimation of the remaining lifespan of a water pump and (3) the multi-condition monitoring and health forecasting for a hydraulic rig. The studies take advantage of the real-time diagnostic sensor data that capture essential operational metrics and performance indicators of the associated with machines. They serve as essential sources for the Gaussian process modelling. Through multi-faceted employments in each scenario, the paper showcases that the Gaussian process is able to generate convincing prognosis embedded with uncertainty estimates for each study.

In the remainder of the paper, the Methodology Section introduces the Gaussian process. the Case studies Section provides three case studies along with their respective results. The Discussion Section is dedicated to the discussions of the case study results. Finally, the Conclusions Section presents the conclusion and the potential future avenues for exploration.

## Methodology

The seminal works such as Rasmussen and Williams (2006), Duvenaud et al. (2011) and Roberts et al. (2013) provided an excellent introduction to the Gaussian process that represents a flexible framework for probabilistic modelling in machine learning and statistics. It is a non-parametric approach to modelling data in the sense that it does not make explicit assumptions about the functional form of the relationship between inputs and outputs. Instead, the method relies on the covariance function to capture the relationships between data points. This allows Gaussian process to adapt to data complexity and make predictions without being constrained by a fixed number of parameters. In essence, a Gaussian process defines a distribution over continuous functions, from which any finite subset of function values collectively follows a multivariate Gaussian distribution.

Effectively, a Gaussian process f(x) can be characterised by a mean function m(x) and a covariance function $k(x,{\;x}^{\prime })$ as formulated:

(1) $$\begin{array}{c} \;f(x)=GP(m(x),\;k(x,\;x^{\prime })) \end{array}$$

where x is a vector composed of the values of the independent variables. In the context of maintenance activities, these variables can represent various sensor readings monitoring the operation of equipment. Effectively, from the viewpoint of Bayesian inference, a Gaussian process delineates two fundamental phases: the prior and the posterior. The prior establishes the foundational assumptions and the domain knowledge that contribute to the distribution of functions. It serves as the fundamental representation of uncertainty over the space of functions before the provision of sensor data. As observations are obtained, for example, the sensor records of the operation and the health status of equipment over a certain period, the prior undergoes a transformative process and moves to the posterior phase. The posterior, encapsulating both prior beliefs and the numerical evidence from observations, provides predictions for the target variable of interest, such as the health status of equipment in future time series, at an infinite number of locations within the parameter space.

To illustrate, define $X_{o}$ as the matrix comprising the observed sensor record vectors, i.e. the entire training inputs, and $y_{o}$ as the vector of the observed target variables in a past time series, respectively. In a similar manner, define $X_{p}$ as the matrix comprising the sensor records belonging to a future time series, i.e. the testing inputs, and $y_{p}$ as the vector of the target variables in that same period. The objective is thus to predict $y_{p}$ and the affiliated uncertainty at the new inputs $X_{p}$ . This can be formulated as conditional Gaussian distributions as shown:

(2) $$\begin{array}{c} \;p(y_{p}|y_{o},\;X_{o},\;X_{p}) \end{array}$$

According to the definition of Gaussian process:

(3) $$\begin{array}{c} y_{o}=f(X_{o})∼N(m(X_{o}),K(X_{o},X_{o})) \end{array}$$

(4) $$\begin{array}{c} y_{p}=f(X_{p})∼N(m(X_{p}),K(X_{p},X_{p})) \end{array}$$

and the joint prior distribution of $y_{o}$ and $y_{p}$ can be formulated as:

(5) $$\begin{array}{c} \left[\begin{array}{c} y_{o}\\ y_{p} \end{array}\right]∼N\left(\left[\begin{array}{c} m(X_{o})\\ m(X_{p}) \end{array}\right],\left[\begin{array}{cc} K(X_{o},X_{o}) & K(X_{o},X_{p})\\ K(X_{p},X_{o}) & K(X_{p},X_{p}) \end{array}\right]\right) \end{array}$$

where $m(X_{o})$ is the mean function of $X_{o}$ , and $m(X_{p})$ is the mean function of $X_{p}$ . $K(X_{o},X_{o})$ is the covariance matrix of the training inputs. $K(X_{o},X_{p})$ is the covariance matrix of the training inputs and testing inputs. $K(X_{o},X_{p})$ = $K(X_{p},X_{o}{)}^{T}$ . $K(X_{p},X_{p})$ is the covariance matrix of the testing inputs. As such, equation (2) can be adapted as:

(6) $$\begin{array}{c} \;p(y_{p}|y_{o},\;X_{o},\;X_{p})∼N({\bar{y}}_{p},C(y_{p})) \end{array}$$

where ${\bar{y}}_{p}$ represents the expected values of $y_{p}$ , and $C(y_{p})$ being the covariance of $y_{p}$ measures the prediction uncertainty. They can be obtained respectively as follows:

(7) $$\begin{array}{c} {\bar{y}}_{p}=m(X_{p})+K(X_{p},X_{o}){\left[K(X_{o},X_{o})\right]}^{-1}(y_{o}-m(X_{o})) \end{array}$$

(8) $$\begin{array}{c} C(y_{p})=K(X_{p},X_{p})-K(X_{p},X_{o}){\left[K(X_{o},X_{o})\right]}^{-1}K(X_{o},X_{p}) \end{array}$$

Two considerations should be noted:

- Unless the prior belief or knowledge is specified, $m(X_{o})$ in practice is often set as 0. This assumption simplifies the modelling process by not estimating an additional parameter. Besides, it ensures the initial neutrality before data observation by not importing prior biases. This augments the flexibility and the interpretability of Gaussian process. This paper holds this assumption.
- If data are deemed noisy, a noise term α should be taken into account. α is independent and identically distributed and follows a Gaussian distribution with the mean being 0 and the variance being $\sigma^{2}$ :
(9) $$\begin{array}{c} \alpha ∼N(0,\sigma^{2}) \end{array}$$

As such, ${\bar{y}}_{p}$ and $C(y_{p})$ will be reformulated as:

(10) $$\begin{array}{c} {\bar{y}}_{p}=m(X_{p})+K(X_{p},X_{o}){\left[K(X_{o},X_{o})+\sigma^{2}I\right]}^{-1}\\ \left(y_{o}-m(X_{o})\right) \end{array}$$

(11) $$\begin{array}{c} C(y_{p})=K(X_{p},X_{p})-K(X_{p},X_{o}){\left[K(X_{o},X_{o})+\sigma^{2}I\right]}^{-1}\\ K(X_{o},X_{p}) \end{array}$$

where *I* is the identity matrix. The noise can be interpreted as the variability or uncertainty in the observed data that cannot be explained by the underlying function being modelled. Higher noise leads to greater deviation of the observed data from the underlying function, resulting in wider confidence intervals (CIs) around the predicted mean values in the Gaussian process model. Conversely, lower noise suggests a closer alignment between the data and the underlying function, reducing uncertainty in the predictions. However, this alignment may lead to poorer model generalisation performance due to underestimation of uncertainty and overfitting to the training data.

In Gaussian process, the covariance function, often known as the kernel function, serves as the bedrock in modelling the similarities between data points, influencing the model's ability to learn and predict patterns. The kernel function defines the shape and characteristics of the covariance matrix, capturing complex patterns and dependencies in the data. By selecting appropriate kernel function(s), engineers can tailor the model to specific problem domains, thereby accommodating various types of structures existent in the maintenance data, such as smooth trends, periodic fluctuations and abrupt changes. It should be noted that Gaussian process allows the utilisation of single or multiple kernels. An individual kernel may already suffice to describe a distinct and prominent pattern, whereas a composition containing two or more kernels will be able to capture intersected and intricate patterns. Hence, Gaussian process demands a careful kernel function selection. Some studies were conducted to optimise this procedure (Abdessalem et al., 2017; Akian et al., 2022; Shadrin et al., 2021; Teng et al., 2020). In addition, Duvenaud (2017) proposed a decent reference for kernel selection. Yet, the mainstream selection technique relates to the experience and knowledge of the practitioner together with a repetitive trial-and-error practice (Cui et al., 2021).

Each kernel function has a group of hyperparameters to be tuned. This is generally done by maximising the log marginal likelihood of the Gaussian process. Define β as a vector of the hyperparameters, $\hat{\beta }$ as a vector of the tuned hyperparameters, *n* is the number of data points and $K_{y_{o}}$ as the covariance matrix of $y_{o}$ , then the maximisation can be denoted by**2**. In (12), there should have been a 'beta' symbol β exactly under 'argmax', as shown in my submitted manuscript. since wer are unable to complete this operation in your system, your help will be highly appreciated.

(12) $$\hat{\beta }={\mathrm{argmax}}_{\beta }\;(\log\;p(y_{o}|X_{o},\beta ))$$

(13) $$\begin{array}{r} \begin{array}{c} \log\;p(y_{o}|X_{o},\beta )=-\frac{1}{2}y_{o}^{T}{\left(K_{y_{o}}+\sigma^{2}I\right)}^{-1}y_{o}\\ -\frac{1}{2}\log\;|K_{y_{o}}+\sigma^{2}I|-\frac{n}{2}\log\;2\pi \end{array} \end{array}$$

In addition to maximising the log marginal likelihood, implementing cross-validation or defining a customised objective function tailored to specific performance metrics, such as minimising mean squared error or maximising classification accuracy, can also be valid alternatives for hyperparameter optimisation in Gaussian process.

## Case studies

In this section, Gaussian process will be implemented in three case studies to demonstrate its effectiveness in predictive maintenance activities. Each of the case studies is elaborated to link to a distinctive task, with the objective of exhibiting the versatility of the proposed approach. In particular, case study 1 serves as a baseline using a synthetic single-sensor dataset, whereas case studies 2 and 3 involve more complicated prediction tasks with multi-sensor datasets collected from real-world sources that are larger and more representative. The studies are conducted primarily using the GPy framework (GPy, 2012) in Python, based on Intel^®^ Core™ i7-9750H CPU at 2.6 GHz processor and 32.0 GB RAM. GPy is a powerful and friendly library implementing Gaussian process modelling, with a comprehensive set of tools that support model customisation, hyperparameter tuning and result visualisation.

### Evaluation metrics

This paper adopts the four metrics formulated in equations (14) to (17) to evaluate the performance of the proposed Gaussian process models. They are commonly used for assessing model outcomes in a prediction task.

(14) $$\begin{array}{c} \mathrm{RMSE}=\sqrt{\frac{1}{n}\overset{n}{\underset{i=1}{\sum }}\;{\left(y_{i}-{\hat{y}}_{i}\right)}^{2}} \end{array}$$

(15) $$\begin{array}{c} \mathrm{MAE}=\frac{1}{n}\overset{n}{\underset{i=1}{\sum }}\;|y_{i}-{\hat{y}}_{i}| \end{array}$$

(16) $$\begin{array}{c} R=\frac{\sum_{i=1}^{n}\;(y_{i}-\bar{y})({\hat{y}}_{i}-\bar{\hat{y}})}{\sqrt{\sum_{i=1}^{n}\;{\left(y_{i}-\bar{y}\right)}^{2}\sum_{i=1}^{n}\;{\left({\hat{y}}_{i}-\bar{\hat{y}}\right)}^{2}}} \end{array}$$

(17) $$\begin{array}{c} R^{2}=1-\frac{\sum_{i=1}^{n}\;{\left(y_{i}-{\hat{y}}_{i}\right)}^{2}}{\sum_{i=1}^{n}\;{\left(y_{i}-\bar{y}\right)}^{2}} \end{array}$$

In the above equations, *n* is the number of data points, $y_{i}$ is the actual target value, ${\hat{y}}_{i}$ is the predicted target value, $\bar{y}$ is the mean of the actual target values and $\bar{\hat{y}}$ is the mean of the predicted target values.

### Kernel function specification

This section lists the kernel functions involved in all three case studies together with respective descriptions of each kernel's characteristic and complexity. These are the kernel functions frequently applied by the practitioners when performing Gaussian process modelling. Notation-wise, *x* and $x^{\prime }$ are two input points; $x-x^{\prime }$ is the Euclidean distance between the two points. *l* is the lengthscale, and σ is the function signal variance. In general, *l* controls the width of the kernel function, whereas σ scales the amplitude of the kernel function. Furthermore, τ is the shape parameter, *p* is the periodicity parameter and *c* is the constant being the intercept of the linear relationship.

*i. Radial Basis Function (RBF) kernel function*
(18) $$\begin{array}{c} k_{\mathrm{RBF}}(x,x^{\prime })=\sigma^{2}\exp\left(-\frac{\left\|x-{x^{\prime }}^{2}\right\|}{2l^{2}}\right) \end{array}$$

Characterised by simplicity, the RBF kernel is adept at modelling smooth and continuous data pattern. The RBF kernel is less computationally demanding compared to more complex kernels. However, its simplicity can sometimes limit its ability to capture intricate patterns in the data.

*ii. Matérn 3/2 kernel function*
(19) $$\begin{array}{r} \begin{array}{c} k_{\mathrm{Mat}e^{\prime }\mathrm{rn}\;3/2}(x,x^{\prime })=\sigma^{2}\left(1+\frac{\sqrt{3}\|x-x^{\prime }\|}{l}\right)\\ \exp\left(-\frac{\sqrt{3}\|x-x^{\prime }\|}{l}\right) \end{array} \end{array}$$

The Matérn 3/2 kernel provides greater flexibility compared to the RBF kernel, which allows for the data patterns with elevated irregularity. The kernel's computational complexity is higher due to more intricate functional form, compared to the RBF kernel.

*iii. Matérn 5/2 kernel function*
(20) $$\begin{array}{r} \begin{array}{c} k_{\mathrm{Mat}e^{\prime }\mathrm{rn}\;5/2\;}(x,x^{\prime })=\sigma^{2}\left(1+\frac{\sqrt{5}\|x-x^{\prime }\|}{l}+\frac{5{\left\|x-x^{\prime }\right\|}^{2}}{3l^{2}}\right)\\ \exp\left(-\frac{\sqrt{5}\|x-x^{\prime }\|}{l}\right) \end{array} \end{array}$$

The Matérn 5/2 kernel exhibits higher flexibility for capturing both smooth trends and irregular patterns compared to the Matérn 3/2 kernel. Yet, its complexity is higher due to the additional terms in its functional form, which introduces an increased number of hyperparameters to optimise. This leads to higher computational effort but also enhances the model's ability to adapt to diverse data patterns.

*iv. Exponential kernel function*
(21) $$\begin{array}{c} k_{\mathrm{Exponential}}(x,x^{\prime })=\sigma^{2}\exp\left(-\frac{\left\|x-x^{\prime }\right\|}{l}\right) \end{array}$$

The exponential kernel enables sharper transitions or abrupt changes in the data pattern compared to the RBF kernel, while its capability to capture smooth and continuous pattern is preserved. Its similarity in functional form to the RBF kernel suggests faster computations relative to more complex kernels such as Matérn. Despite these advantages, the exponential kernel's simplicity may limit its ability to comprehensively model complex data relationships.

*v. Rational quadratic (RatQuad) kernel function*
(22) $$\begin{array}{c} k_{\mathrm{RatQuad}}(x,x^{\prime })=\sigma^{2}{\left(1+\frac{{\left\|x-x^{\prime }\right\|}^{2}}{2\tau l^{2}}\right)}^{-\tau } \end{array}$$

The RatQuad kernel is a scale mixture of RBF kernels with different lengthscales that provides a versatile tool for capturing various degrees of smoothness in the data. The additional shape parameter τ controls the relative weighting of the lengthscales, thereby influencing the degree of differentiability. This increases the kernel's complexity and the computational burden of hyperparameter optimisation.

*vi. Periodic kernel function*
(23) $$\begin{array}{c} k_{\mathrm{Periodic}}(x,x^{\prime })=\sigma^{2}\exp\left(-\frac{2\sin^{2}(\pi (\|x-x^{\prime }\|/p))}{l^{2}}\right) \end{array}$$

The periodic kernel introduces periodicity into the covariance function with the aim of modelling cyclic patterns. The hyperparameter *p* influences the length of the periodic cycles. The periodicity of this kernel introduces complexity due to the need to optimise for cyclic behaviours, which can be computationally intensive.

*vii. Linear kernel function*
(24) $$\begin{array}{c} k_{\mathrm{Linear}}(x,x^{\prime })=\sigma^{2}(x-c)(x^{\prime }-c) \end{array}$$

The linear kernel captures linear relationships in data, characterised by its simple design which results in lower computational complexity compared to other kernels. However, its efficacy in capturing non-linear patterns is limited.

### Case study 1: temporal prediction of sensor data degradation trends

Case study 1 focuses on predicting the degradation trends of a specific component critical to the machine's functionality. Presumably, the component's condition is continuously monitored by a sensor installed. The sensor data is intentionally designed to encompass two principal patterns:

- *Periodical repetition pattern with increasing amplitude:* This pattern reflects the recurring nature of certain operational behaviours within the equipment. These repetitions may signify predictable operational fluctuations or periodic occurrences relevant to the operation. Meanwhile, the amplitude of the periodic variations intensifies as time progresses, which indicates a heightened impact of operational fluctuations.
- *Gradual-ascending pattern:* This pattern demonstrates a global increase in sensor readings over time. Such a trend could indicate progressive wear and tear or the evolving operational state of the monitored equipment component.

Additionally, the data incorporate some oscillations to simulate the variability encountered in real-world sensor records. The data's characteristics are representative of multiple equipment scenarios used in mining sector, each with its unique operational behaviours and degradation patterns. For instance:

*Scenario 1: monitoring the bearing temperature of a crusher*. In this scenario, the focus lies on monitoring the temperature of the bearings within a crusher. The sensor therefore captures changing bearing temperatures that reveal two predominant patterns: an overall rise attributed to gradual wear due to prolonged use and periodic fluctuations corresponding to the operational cycles of the crusher machine. The cyclical increase in amplitude could signify higher temperature fluctuations due to (1) the addition of harder materials requiring heightened force to crush, (2) the inadequate lubrication or material-wise deterioration, (3) extended operational hours imposing additional stress on the machine and (4) heightened ambient temperatures exacerbating frictional heating within the bearing. The oscillation within the data can be explained by operational or ambient temperature fluctuation that influences the sensor recording process.

*Scenario 2: monitoring the vibration of the suspension of a haulage truck*. In this scenario, the focus is on monitoring the vibration levels within the suspension system of a mine haulage truck using a vibration sensor. The gradual increase in vibration levels originates from long-term wear and tear on the system. Simultaneously, the periodic variations correspond to the operational cycles of the truck, wherein vibrations fluctuate in synchrony with the truck's movements, loading and unloading activities (periodical repetition pattern). The increase in periodical amplitude may be attributed to heavier loads, deteriorating road conditions or cumulative friction, mechanical stress and material fatigue on the suspension components, which lead to more pronounced vibrations during operational cycles. The oscillation in the data overtime may stem from factors such as variations in road conditions or mechanical issues within the suspension system, which leads to fluctuations in the observed vibration levels.

**Figure 1.**
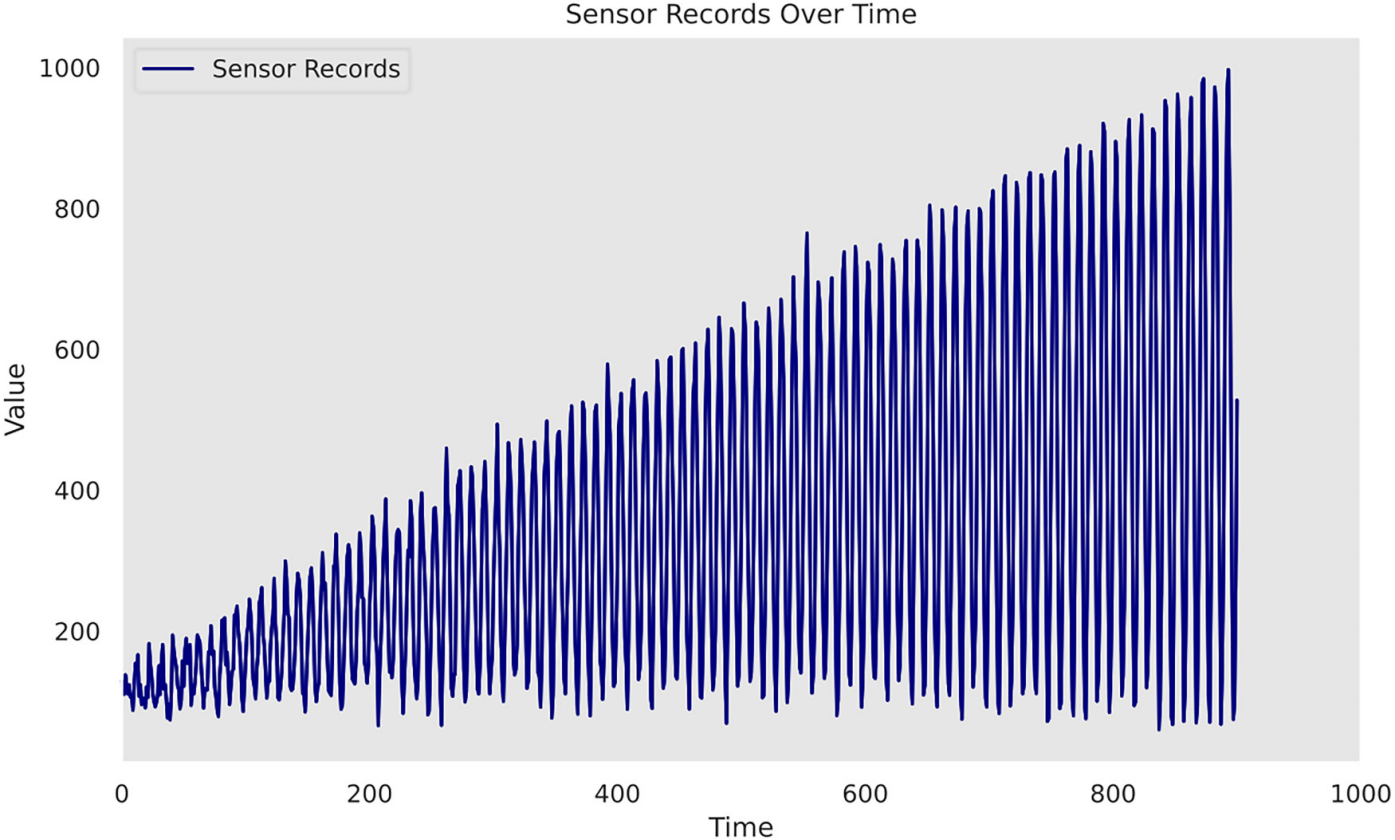
Time series sensor records of case study 1.

Figure 1 illustrates the simulated data used in case study 1. The dataset is composed of 1000 timesteps and corresponding sensor readings. As the dataset is hypothetic, it is important to note that the time and sensor values in the study do not directly correspond to specific units or measurements but are for demonstrative purposes. Instead, they are characterised by their scalability, which allows them to accommodate a wide range of data sizes and dimensions. The training set is composed of the first 700 records; the idea is to train a proper Gaussian process model by learning the inherent patterns. Thereafter, the following 200 records constitute the validation set and will be used to evaluate the model performance. As a further step, the optimal model from the validation step will be selected and used to conduct predictions on the last 100 timestamps. The goal is to validate the Gaussian process's ability to generate meaningful prognosis in the long term, especially in the presence of missing sensor records due to its malfunction. As such, no sensor values on these 100 units are provided in the original dataset. Due to the existence of multiple salient patterns in the data, the utilisation of composite kernel scheme is considered for case study 1 to model cyclic repetitions and address varying amplitudes and irregularities presented. The hyperparameters of the kernel functions are optimised defaulting to the GPy's limited-memory Broyden–Fletcher–Goldfarb–Shanno (L-BFGS) algorithm. Being a gradient-based optimisation technique, it aims to maximise the log marginal likelihood to find the optimal parameters that best fit the observed data. Optimisation bounds for period and noise variance are defined specifically in Table 1. The remaining hyperparameters are left unconstrained. However, to maintain positivity during the optimisation process, ‘model.constrain_positive()’ is employed. The target value is normalised during the optimisation by setting ‘Normalizer = True’ in GPy. The performance of three Gaussian process models on the 200-sample validation set is quantified in Table 2.

**Table 1.** Hyperparameter optimisation bounds in case study 1.

Hyperparameters	Bounds
Period	[5, 15]
Noise variance	[0.05, 0.3]

**Table 2.** Three kernel designs and their respective performance on the validation set of case study 1.

Kernel	RMSE	MAE	$R^{2}$	R
Periodic × linear + RBF	24.873	20.394	0.993	0.997
Periodic × linear + Matérn 5/2	21.576	17.436	0.995	0.998
Periodic × linear + exponential	26.610	21.974	0.992	0.998

**Figure 2.**
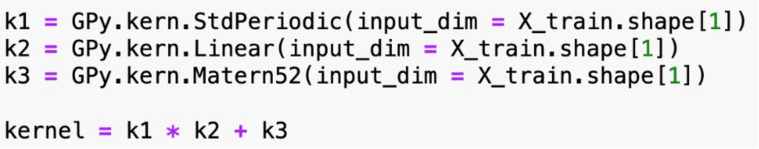
Composite kernel design for case study 1.

**Figure 3.**
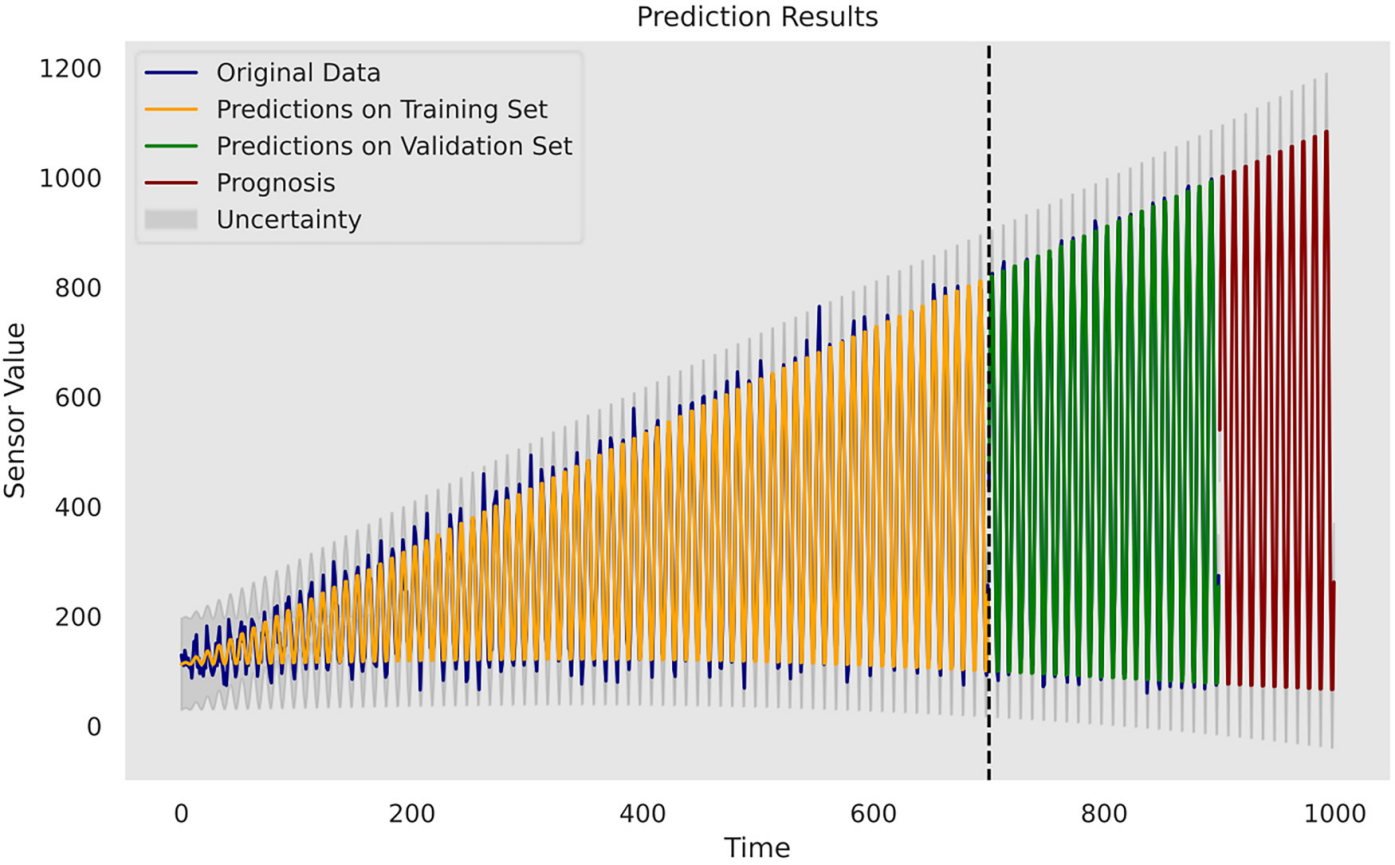
Prediction and the extrapolation with uncertainty on the last 100 time units.

Upon evaluation, it is determined that the model incorporating a periodic kernel, a linear kernel and a Matérn 5/2 kernel exhibits the most favourable results on the designated set and will be used for the prognosis task. Figure 2 depicts the construction of this composite kernel using GPy. Figure 3 plots the complete model inference outcomes using the proposed composite kernel on the dataset. The grey-shaded areas represent the uncertainty estimates of the model quantified in a 95% CI.

### Case study 2: machine remaining useful life prediction

The objective of case study 2 is to demonstrate the potential of Gaussian process in forecasting the remaining lifespan of the mining machinery. More commonly known as remaining useful life (RUL), this metric is the residual time for a piece of machinery to perform its desired functionality before the failure appears. RUL estimation plays an important role in the predictive maintenance programme since it provides proactive insights into the tear-and-wear trajectory of a machinery. As such, this task provides a quantitative support for maintenance personnel with respect to the maintenance planning and cost reduction. Following the prediction, the critical repair and replacement would be executed in advance to minimise production loss and mitigate safety hazards, while unnecessary or emergent repairs can be avoided.

The study is based on an open dataset that relates to a water pump (Pump_Sensor_Data, 2019). While not originally sourced from a mining water pump, the dataset is representative of the operational dynamics encountered in such equipment. The dataset is composed of a collection of temporal observations, with each sample documenting real-time sensor readings capturing diverse operational parameters of the pump. These sensor values serve as the input features for the analysis. The target variable is encoded as a binary, indicating the presence of the failure events.

In effect, a water pump plays a crucial role in mining operations, such as water supply for processing, backfill surpassing or cooling equipment from a source to mine site or dewatering underground openings from underground to surface. The harsh environment of the underground worksite deteriorates the longevity and reliability of the pump, which would impact the safety of the miners and diminish their productivity. As such, it is imperative to maintain the pump in its optimal condition. Modern water pumps for mining operations are usually equipped with advanced sensor technology. Some related research can be found in Wang et al. (2009), Wu and Chen (2011) and Brodny and Tutak (2022). The sensors are strategically integrated into pump systems to continuously monitor various parameters such as water pressure, water level and flow rate. By leveraging real-time data insights provided by these sensors, operators can obtain valuable information about the health and performance of the pumps, which enables them to prognostically notify the maintenance unit to perform repair and maintenance.

The original dataset contains 51 sensors as inputs and 166 441 records. The binary output column indicates that, in total, seven failures are observed across the entire time series. As the goal is to perform the predictive maintenance by forecasting the RUL of the pump, an important pre-process step is to create a new column used to record the remaining time to failure. Each remaining time is computed according to the given timestamp affiliated to each sample. Hence, the pump under investigation has seven complete run-to-failure life cycles. According to data analysis, sensor_15, sensor_50 and sensor_51 contain a high percentage of missing values (100.000%, 15.215% and 8.144%, respectively), whereas the missing value percentages for the rest sensors are lower than 0.200%. Thus, the columns corresponding to the three sensors are discarded, and the rest missing values are interpolated by the median of each related column.

A critical step is then to adapt the timestamp into a representative form. Specifically, each sample is recorded in seconds according to the timestamp values in the original dataset. However, in real life it is not necessary to forecast the RUL of the machine with second-level precision. A larger precision level allows for a more pragmatic approach to RUL monitoring and estimation, and it enables maintenance teams to identify equipment degradation over longer time horizons, thus facilitating more informed decision-making and predictive maintenance strategies. In addition, this approach reduces the computational complexity of the analysis. As such, a key process is to agglomerate the original second-level timestamp intervals into hourly intervals. Consequently, this transformation reduces the sample size to 2541.

In the remainder of the workflow, an important practice is to properly build the training and testing data. Different from most machine-learning works, a random train–test split is not applicable as it fails to respect the inherent temporal order of the sensor data, which is crucial for capturing the sequential dependencies. As such, this case study considers employing the first six consecutive run-to-failure cycles to train and validate the model, whereas the model performance will be evaluated in the last 60 h of the last cycle instead of the full cycle. This is because the later period of the machine is often of the major concern of the maintenance crews as the machine approaches failure rapidly. A similar viewpoint can be found in Zeng et al. (2021).

(25) $$\begin{array}{c} x_{\mathrm{normalized}}=\frac{x-x_{\min}}{x_{\max}-x_{\min}} \end{array}$$

Due to the sensor values spanning vastly different ranges, the input features are normalised into [0,1] range using min–max normalisation following equation (25), where *x* is the original value, $x_{\mathrm{normalized}}$ is the normalised value, $x_{\min}$ is the minimum value of the feature and $x_{\max}$ is the maximum value of the feature. With respect to the target variable, the empirical testing discloses that the implementation of QuantileTransformer from scikit-learn (Pedregosa et al., 2011) is deemed suitable for this task. The output distribution of the target values is selected as ‘uniform’. The modelling process will be based on the transformed target values, followed by an inversion to render the prediction results comprehensible.

**Figure 4:**
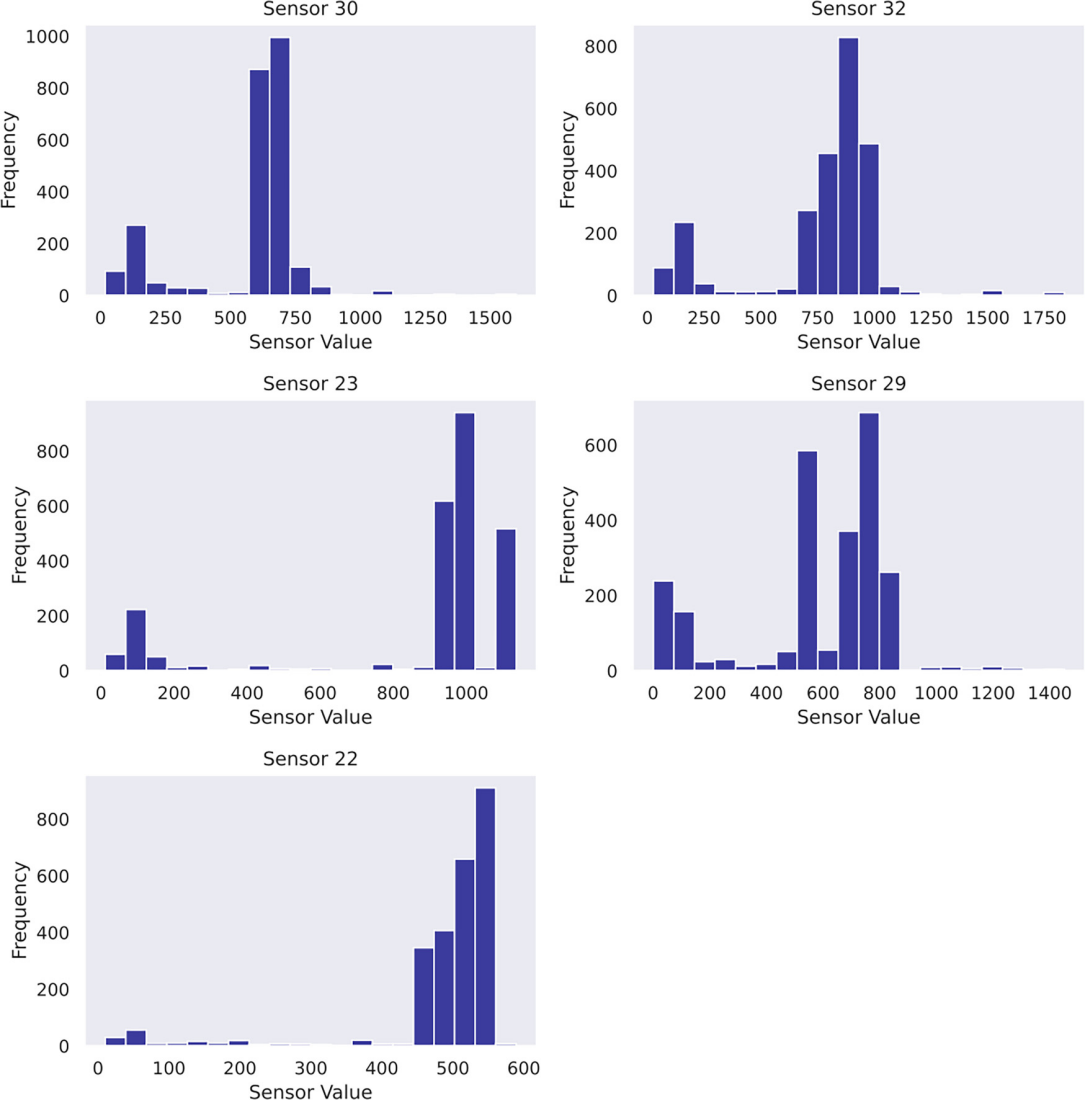
Histograms of selected features in case study 2.

**Figure 5:**
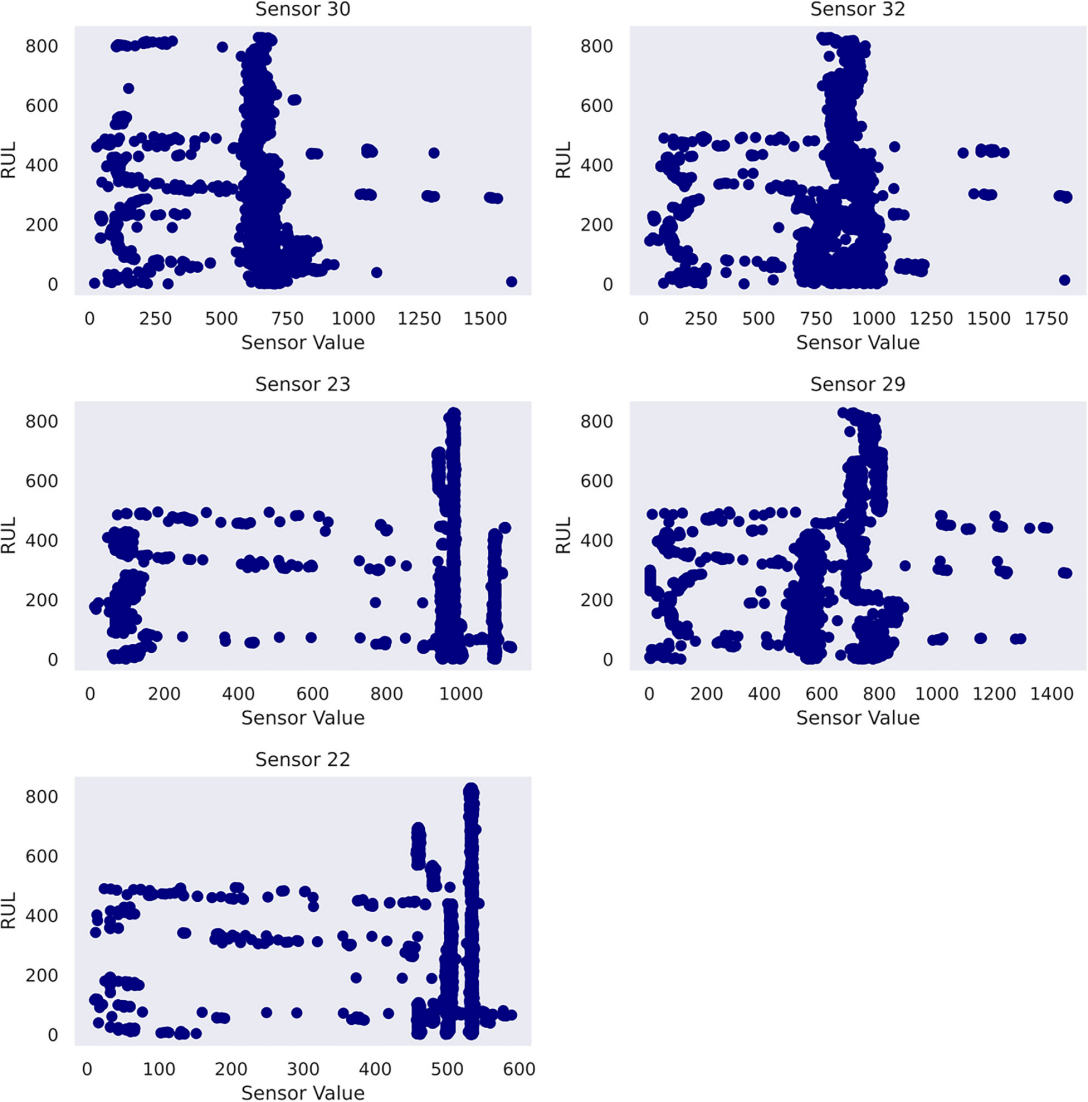
Scatter plots of selected features in case study 2.

Another practice is to perform a feature selection for the given dataset. It serves (1) identifying the most crucial sensors that best explain the RUL diminution, and (2) reducing the input dimensionality to elevate computation efficiency. This study designs a feature selection pipeline based on random forest regressor and mutual information scores imported from scikit-learn (Pedregosa et al., 2011). The top features are selected based on a combined ranking mechanism achieved by adding normalised feature importance and mutual information scores element-wise to obtain combined rankings. Five features have proven suitable after iterative trials. As a result, sensor_30, sensor_32, sensor_23, sensor_29 and sensor_22 are determined as the input features to Gaussian process usage in case study 2. To gain insight into the data, the sensor histograms are shown in Figure 4, whereas the scatter plots showing the correlation between the selected sensors and the pump's RUL are shown in Figure 5.

Before prediction, a hold-out validation analysis is conducted on the training data, on which different kernel configurations and their ensuing performance are assessed. The objective is to determine the most suitable kernel function. The validation set is composed of a randomly selected 40 continuous samples from the training set. The validation is performed utilising Bayesian optimisation through GPyOpt (The GPyOpt authors, 2016). Bayesian optimisation efficiently navigates search spaces by leveraging prior knowledge and iteratively updating a probabilistic model to select the next best candidate for evaluation, thereby minimising computational expense and maximising performance gains. The objective function is to minimise the root mean square error (RMSE) on the validation data. The bounds for hyperparameter optimisation involved in the search are specified in Table 3.

**Table 3.** Hyperparameter optimisation bounds in case study 2.

Kernels	Hyperparameters	Bounds
RBF, Matérn 3/2, Matérn 5/2, exponential, RatQuad	Lengthscale	[0.01, 0.1]
	Signal variance	[0.01, 0.1]
	Shape	[0.01, 3]
	Noise variance	[0.04, 0.06]

**Figure 6.**
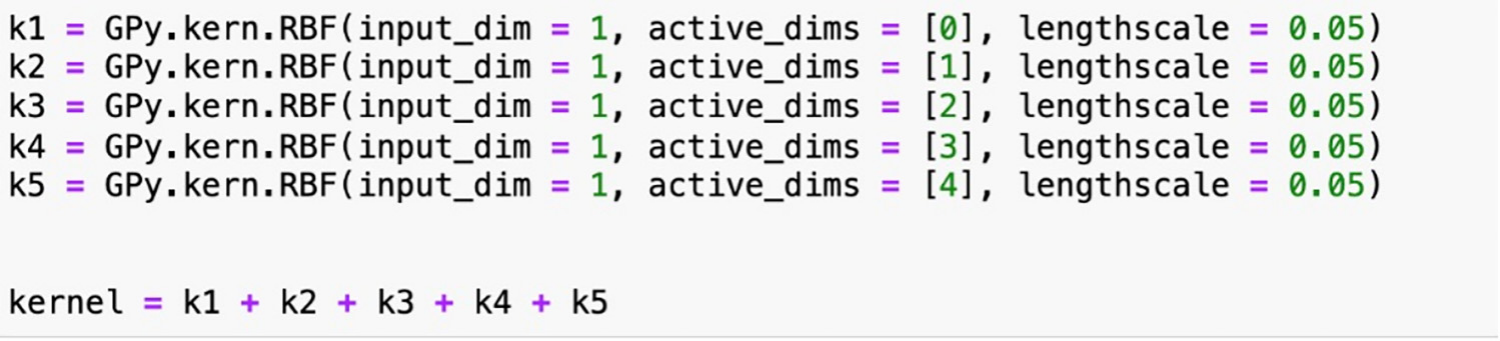
RBF kernel design of case study 2 as an example.

The performance of evaluated kernels on the validation set is presented in Table 4. Results indicate that RBF, exponential and Matérn 3/2 kernels achieve the top three lowest RMSE values on the validation set. Consequently, these three kernel designs are preferred over others for the subsequent prediction task. Furthermore, to explore the optimal model performance, two new composite kernels, RBF + Matérn 3/2 and Exponential + Matérn 3/2, are developed. Given that the dataset has five sensor features, that is, five input dimensions, five sub-kernels are first applied to each individual dimension and the final kernel is the summation of the sub-kernels from each dimension. For example, the RBF kernel for the testing phase is shown in Figure 6. Each model is built on the complete training set, optimised by GPy's L-BFGS algorithm, and implemented on the 60-h testing data. The hyperparameter bounds remain the same as in the validation process except that the constraint for the signal variance is transitioned to solely maintaining positivity. This allows the model to adapt elevated flexibility to the data and can potentially improve model fit. Furthermore, a prior value of 0.05 is set for lengthscale after careful trials.

**Table 4.** Results of different kernels on the validation set in case study 2.

Kernel	RMSE
RBF	24.554
Matérn 3/2	25.141
Matérn 5/2	30.693
Exponential	24.520
RatQuad	28.450

**Figure 7.**
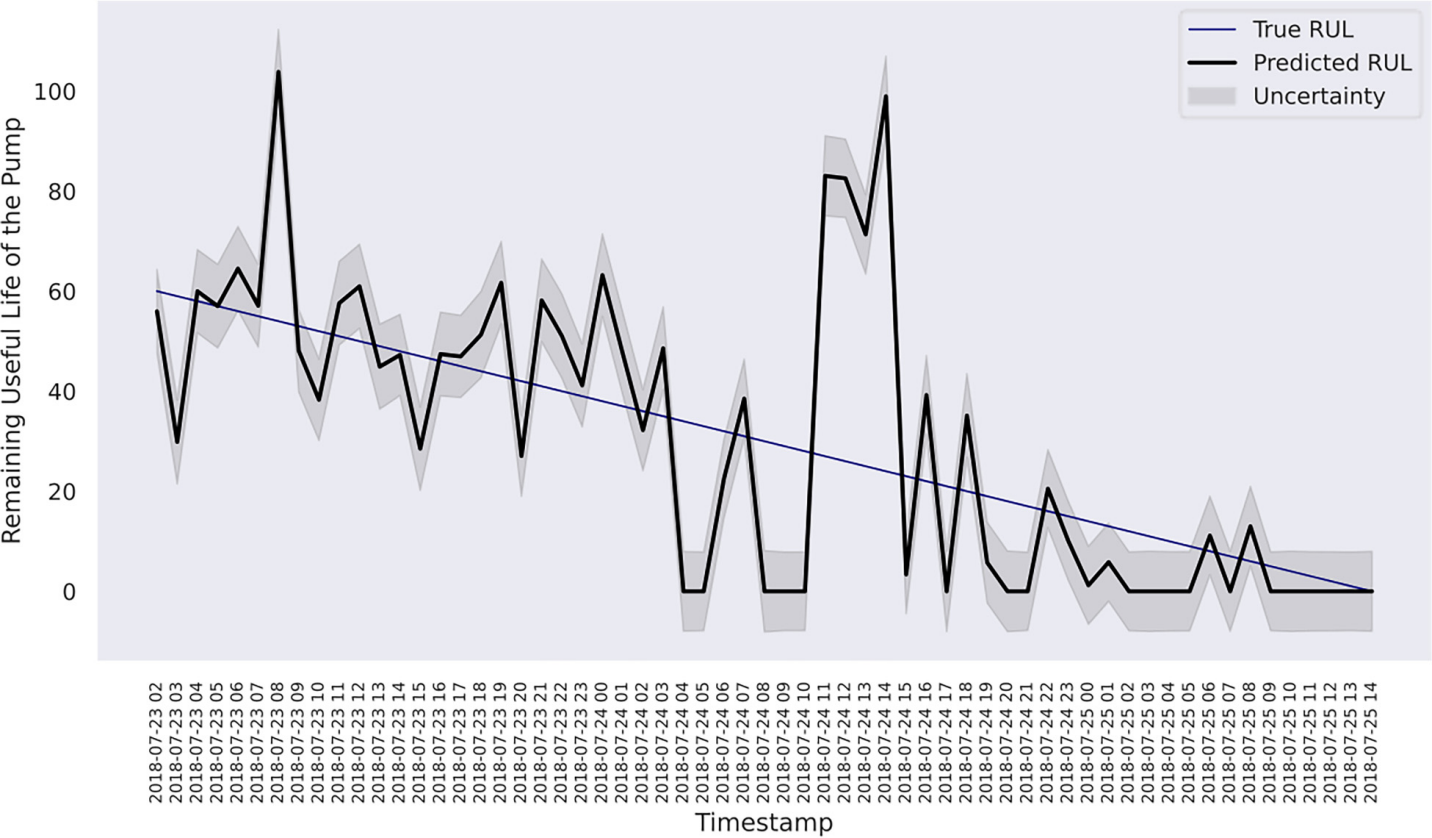
RUL estimation with uncertainty for the last 60 h of the pump using composite exponential + Matérn 3/2 kernels.

**Figure 8.**
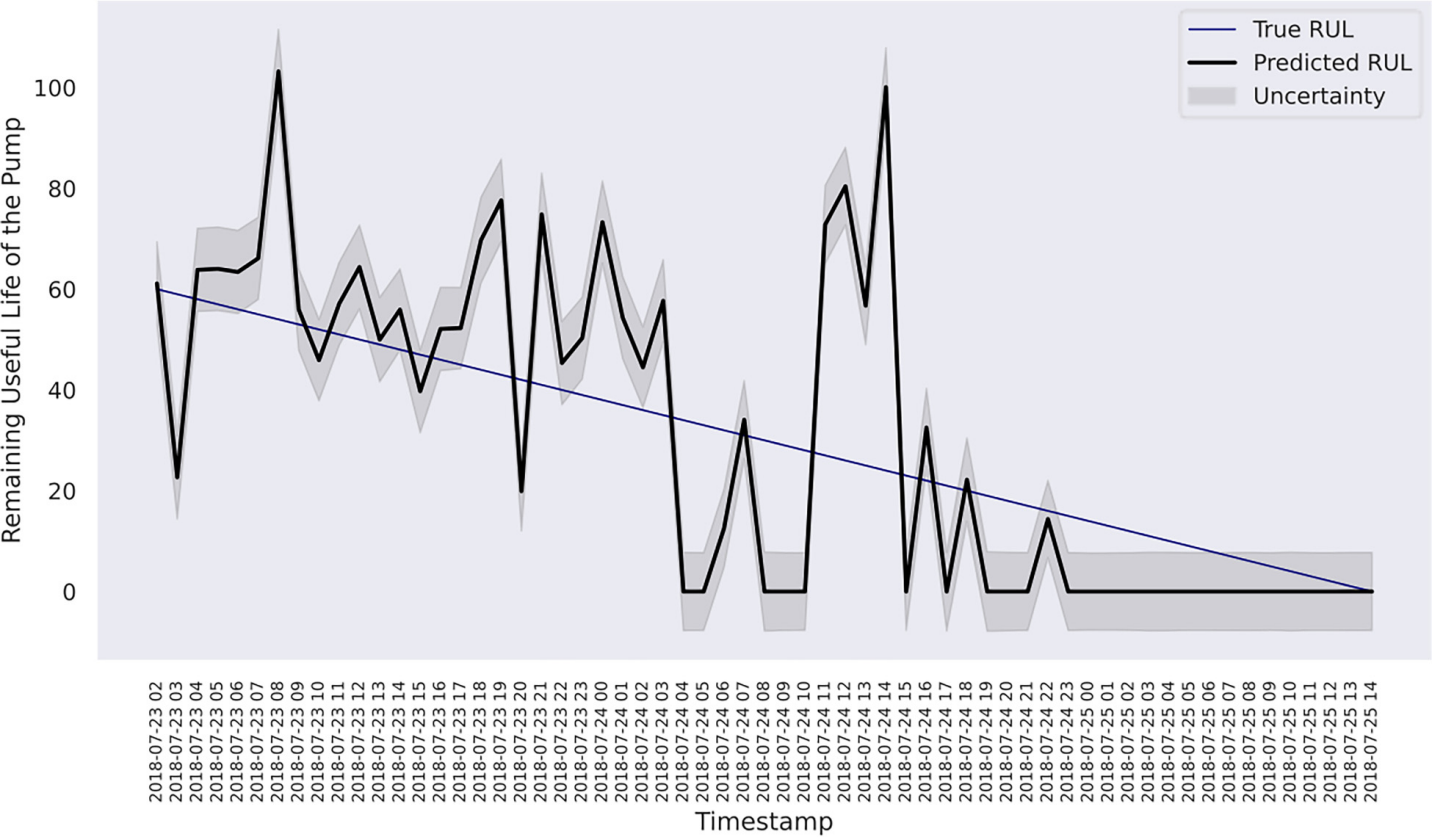
RUL estimation with uncertainty for the last 60 h of the pump using RBF kernel.

The results of each model on the last 60 h data are presented in Table 5. The model with the composition of Matérn 3/2 and exponential achieves the lowest RMSE and MAE , together with the second highest $R^{2}$ and *R*. Meanwhile, the model with an RBF kernel is taken into account due to its leading results in $R^{2}$ and *R*. Hence, both models are selected for further analysis. Figure 7 shows the predicted RUL of the pump and the uncertainty estimates within a 95% CI associated with the composite kernel-based model, whereas the result of RBF kernel-based model is shown in Figure 8.

**Table 5.** Prediction performance of each model on the last 60 h of the testing set.

Kernel	RMSE	MAE	$R^{2}$	R
RBF	22.396	16.570	0.491	0.719
Matérn 3/2	22.293	15.602	0.344	0.595
Exponential	22.068	15.835	0.235	0.521
RBF + Matérn 3/2	22.378	16.131	0.266	0.561
Exponential + Matérn 3/2	21.498	14.958	0.443	0.669

### Case study 3: machine multi-condition monitoring and prediction

Case study 3 applies Gaussian process in sensor-driven machinery condition monitoring and prediction. A public experimental time series dataset (Helwig et al., 2015) is used. The dataset is associated with the operation of a hydraulic rig. The hydraulic rig is a key machinery in mining operations. For instance, drill rigs equipped with hydraulic systems are essential for drilling blast holes and exploration holes in the mining process. The hydraulic power facilitates the rotation and percussion required for drilling through tough rock formations. During the long-time operation, the system will be subjected to stress and strain that may lead to issues such as fluid leakage, pump malfunction or hydraulic cylinder failures. Thus, with continuous monitoring of crucial parameters such as hydraulic pressure, temperature and vibration through sensors integrated into the hydraulic systems, maintenance teams can obtain and analyse voluminous real-time data to identify potential issues and impending failures proactively.

The dataset is appropriate to demonstrate the Gaussian process since it includes a collection of sensor data monitoring in real-time a diverse array of operational parameters which are critical to the performance and safety of the rig under examination. In addition, different from the previous case studies, the dataset represents a multi-condition monitoring and prediction task, as the sensors are associated with four operational conditions of the hydraulic rig followed by a binary column recording the machine stability. The dataset is described in Table 6.

**Table 6.** Dataset description of case study 3.

Number of records: 2205				
Sensor	Physical quantity		Unit	
PS1	Pressure		bar	
PS2				
PS3				
PS4				
PS5				
PS6				
EPS1	Motor power		W	
FS1	Volume flow		L min^−1^	
FS2				
TS1	Temperature		°C	
TS2				
TS3				
TS4				
VS1	Vibration		mm s^−1^	
CE	Cooling efficiency (virtual)		%	
CP	Cooling power (virtual)		kW	
SE	Efficiency factor		%	
Target conditions				
Cooler condition (%)	3: close to total failure	20: reduced efficiency	100: full efficiency	
Valve condition (%)	100: optimal switching behaviour	90: small lag	80: severe lag	73: close to total failure
Internal pump leakage	0: no leakage	1: weak leakage	2: severe leakage	
Hydraulic accumulator (bar)	130: optimal pressure	115: slightly reduced pressure	100: severely reduced pressure	90: close to total failure
Stable flag	0: conditions were stable		1: not reach static conditions	

**Figure 9:**
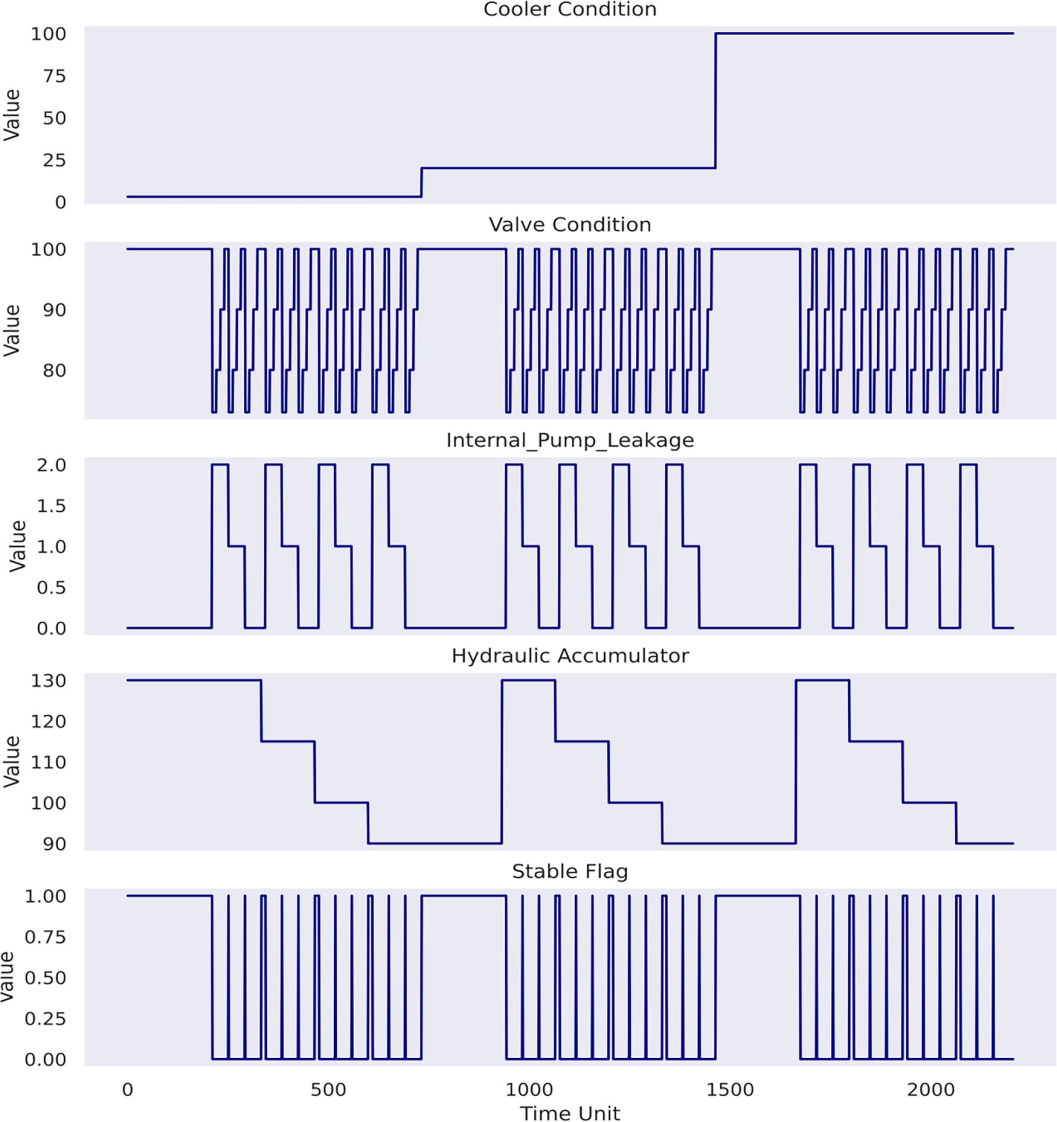
Plots of target variables.

Two considerations of this study are as follows:

- The study will primarily focus on the prognosis of (1) valve condition, (2) internal pump leakage and (3) hydraulic accumulator condition. In effect, according to Figure 9, cooler condition in this dataset lacks discernible periodicity across the entire time series, whereas stable flag only provides an overview of machine status without sufficient granularity and specificity to capture the machine health variations over time. Conversely, the chosen three conditions exhibit salient quantitative variations that warrant attention for effective predictive maintenance strategies.
- The study will predict machine conditions as specific values instead of building a classification problem to resolve, despite the presence of distinct values for each chosen target variables. In fact, all target conditions, excluding stable flag, represent continuous degradation processes over time instead of distinct categorical representation (Helwig et al., 2015). Thus, this strategy allows for capturing the dynamic evolution of each target condition as well as subtle variations and trends.

**Figure 10:**
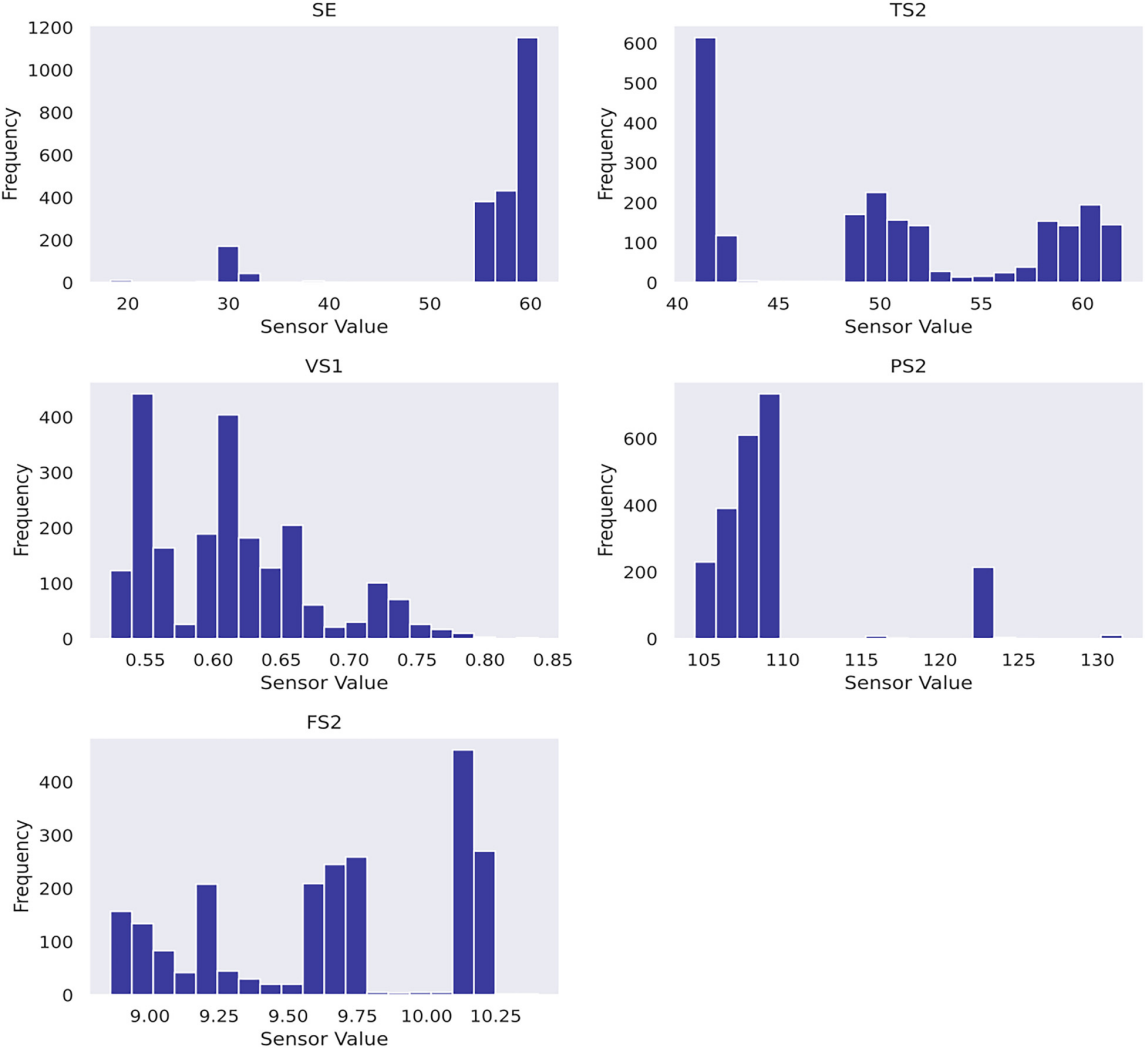
Histogram of selected features of case study 3.

**Figure 11.**
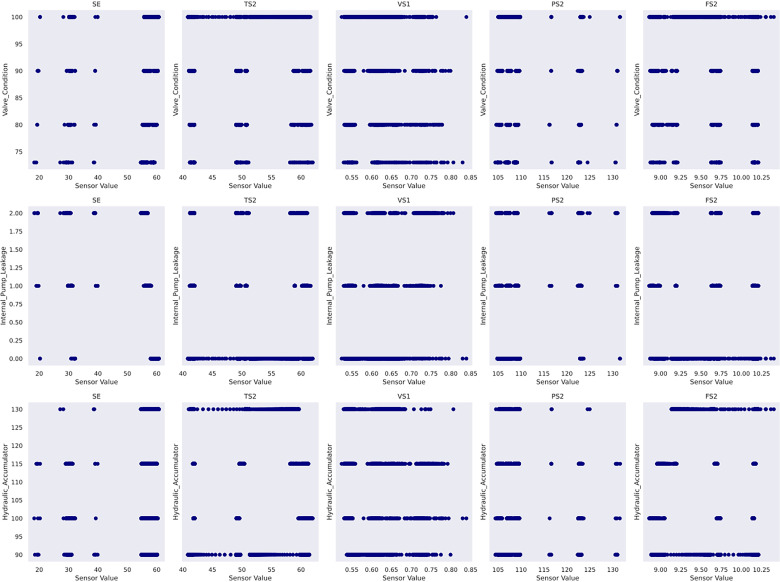
Scatter plots of selected features of case study 3.

The train–validation–test split must respect the chronological order in the given data. As such, the dataset is sequentially divided based on time, with the first 70% allocated for training, followed by 15% for validation and the remaining 15% for testing. The dataset has no reported missing values. In terms of feature selection process, case study 3 develops a varied pipeline compared with that in case study 2. The feature scores from random forest regressor and mutual information scores are weighted and combined using a weighted average approach. As a result, a weight of 0.800 is assigned to the feature importance from the random forest regressor, whereas those from the mutual information approach receive a weight of 0.200 after repetitive experimentation. Next, a threshold is applied to retain the five most influential features. In consequence, SE, TS2, VS1, PS2 and FS2 sensors are selected. Figure 10 shows their histograms, and Figure 11 shows their scatter plots with respect to each of the target conditions to glean knowledge from the data. The features are standardised into [−1, 1] range following equation (26) before Gaussian process modelling, where *x* is the original value, $x_{\mathrm{standardized}}$ is the standardised value, $x_{\mathrm{mean}}$ is the mean of the feature and sd is the standard deviation of the feature:

(26) $$\begin{array}{c} x_{\mathrm{standardized}}=\frac{x-x_{\mathrm{mean}}}{\mathrm{sd}} \end{array}$$

In case study 3, the model is established through GPy. Only one Gaussian process model is built to output simultaneously the predictions of the three target rig conditions. The kernel selection and the hyperparameter optimisation are realised using Bayesian optimisation via GPyOpt. In this workflow, a kernel function is initially selected, and Bayesian optimisation is conducted on the training set to discover the optimal hyperparameters within a pre-defined search space. Next, the model’s performance is assessed on the validation set using the optimised hyperparameters. The optimisation is repeated, if necessary, to find the hyperparameters that result in the best performance on the validation set. This study implements an objective function that is to maximise the average $R^{2}$ . After evaluating multiple kernel functions through this process, the optimal kernel function and its corresponding optimal hyperparameters are determined. The final model is instantiated with the identified optimal kernel function and hyperparameters, and its performance is evaluated on the testing set to gauge its generalisation capability. This systematic approach ensures thorough exploration and selection of the best-performing model configuration for Gaussian process regression. Table 7 presents the hyperparameter search bounds used in the study. Table 8 presents the averaged model performance with different kernels on the validation set.

**Table 7.** Hyperparameter optimisation bounds in case study 3.

Kernel	Hyperparameters	Bounds
RBF, Matérn 3/2, Matérn 5/2, exponential, RatQuad	Lengthscale	[0.5, 8]
	Signal variance	[0.5, 3]
	Shape	[0.5, 3]
	Noise variance	[0.01, 0.2]

**Table 8.** Average $R^{2}$ of different kernels on the validation set of case study 3.

Kernel	Average $R^{2}$
RBF	0.371
Matérn 3/2	0.267
Matérn 5/2	0.394
RatQuad	0.494

**Figure 12.**
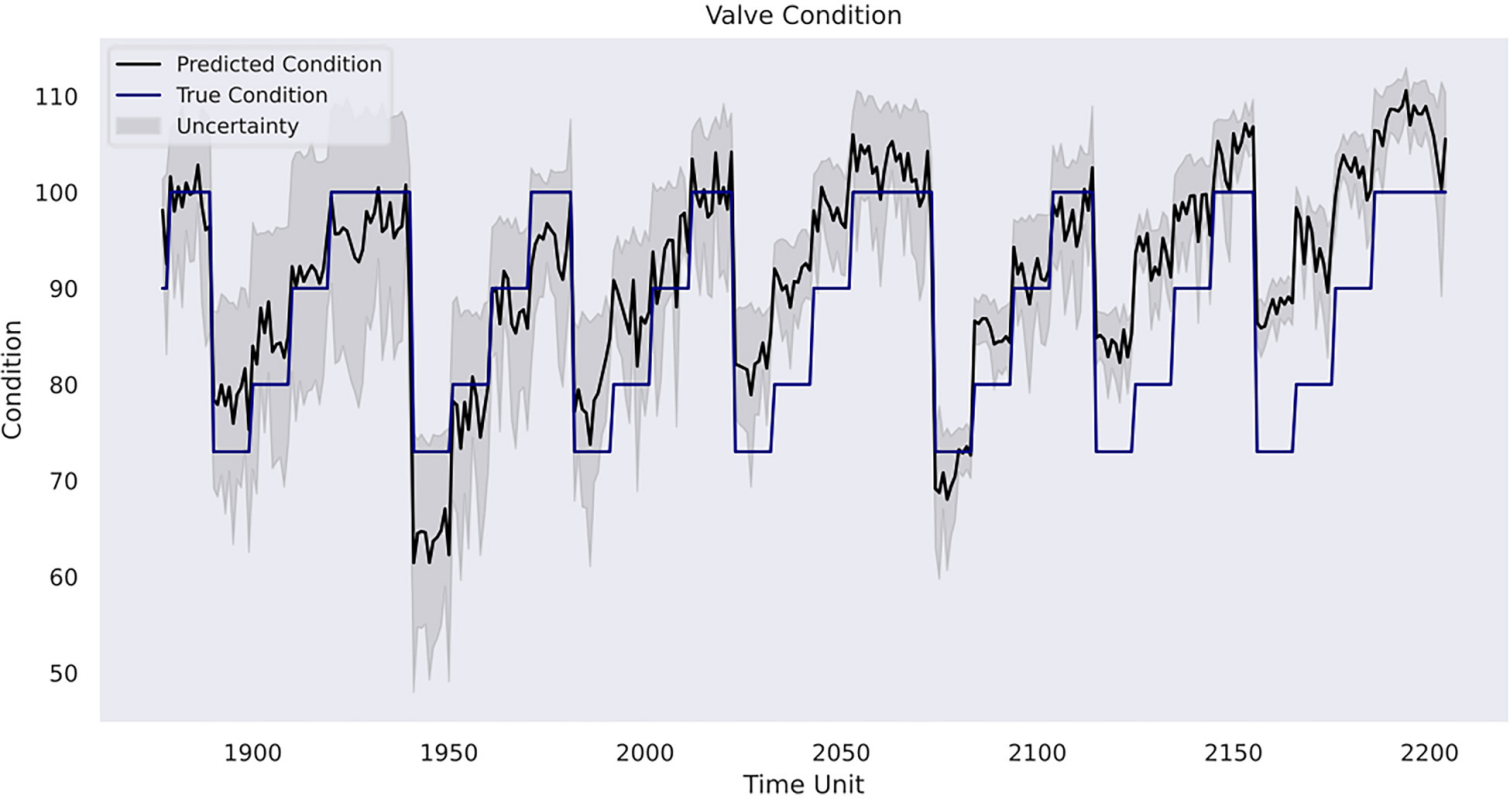
Model prediction on valve condition.

**Figure 13.**
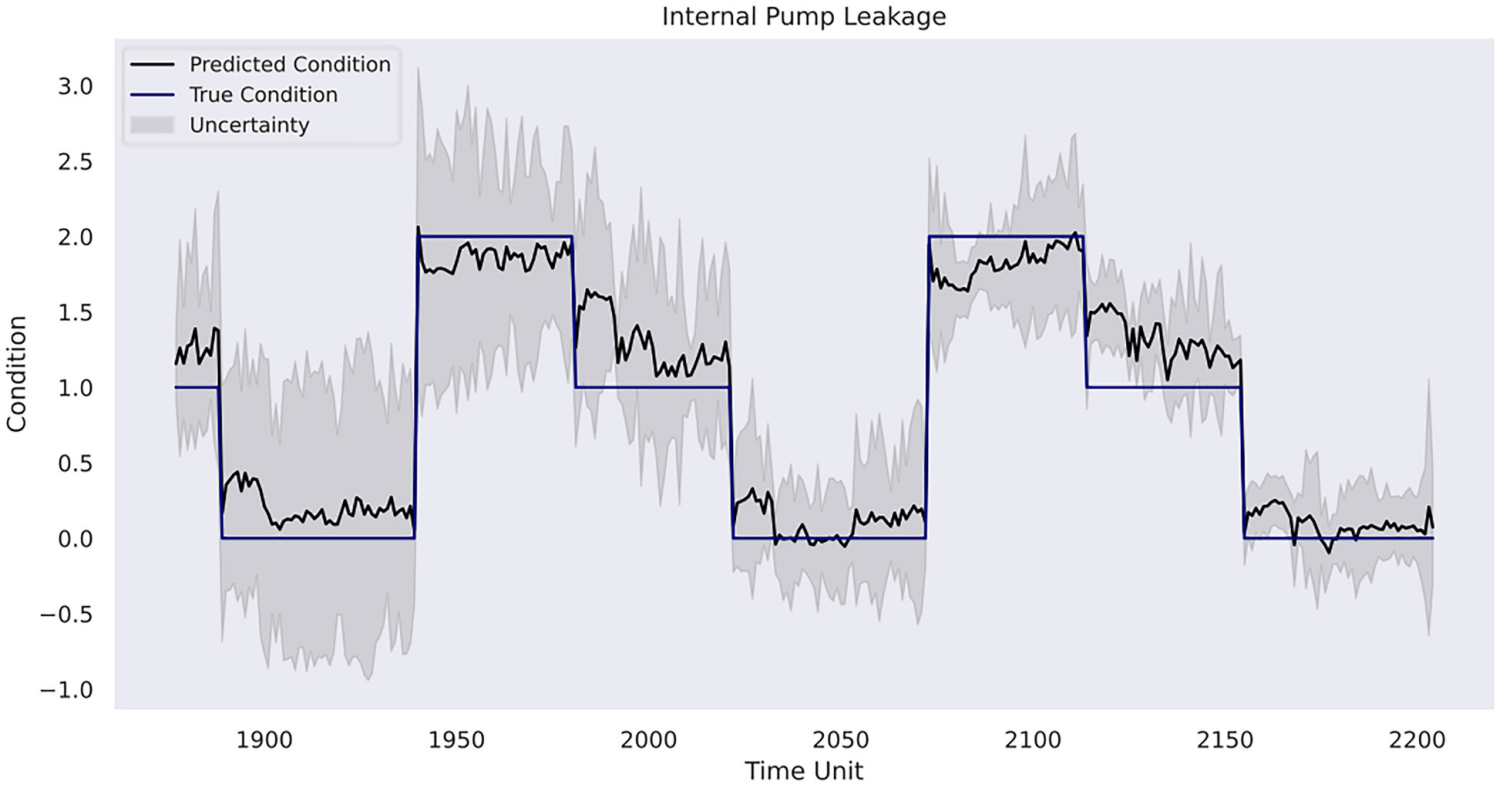
Model prediction on internal pump leakage.

**Figure 14.**
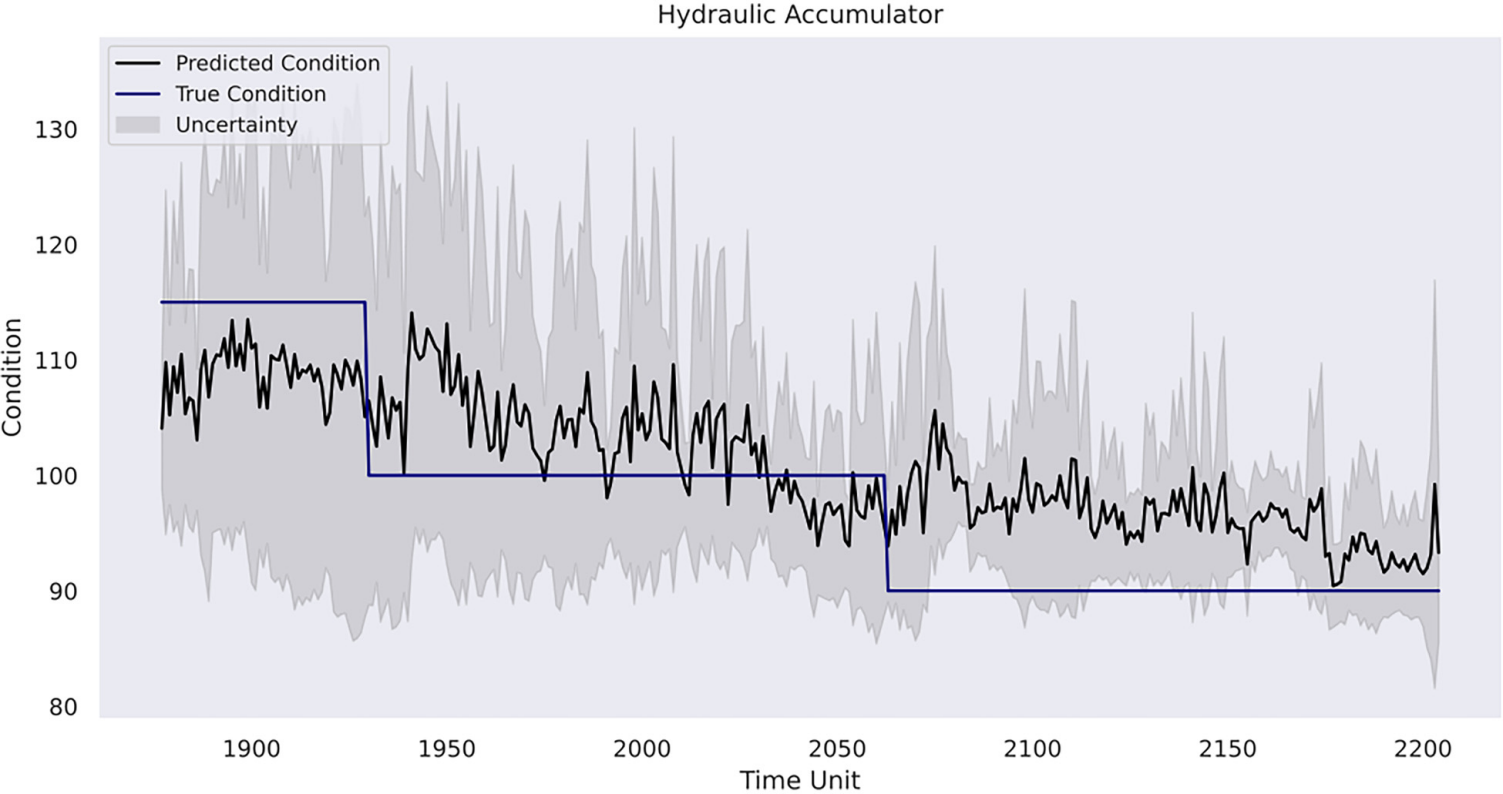
Model prediction on hydraulic accumulator.

RatQuad is selected as the optimal kernel function due to its highest average $R^{2}$ value on the validation set. With the optimised hyperparameters, the result of the corresponding Gaussian process model on the testing set is presented in Table 9. In addition, Figures 12–14 depicts the model predictions on each of the three rig operation conditions and the affiliated uncertainties with a 95% CI compared with the ground truth condition values.

**Table 9.** Performance of the model using RatQuad kernel on the testing set of case study 3.

Target variable	RMSE	MAE	$R^{2}$	R
Valve condition	7.499	6.171	0.504	0.816
Internal pump leakage	0.233	0.190	0.919	0.969
Hydraulic accumulator	6.396	5.641	0.462	0.775
Average performance	4.709	4.001	0.628	0.853

## Discussion

The case studies demonstrate the application of Gaussian process for predictive maintenance tasks. Each case study addresses specific challenges that would be encountered in mining equipment operations. In the first case study, the focus lies on the expected machine progressive wear and tear through the analysis of sensor data trends. The second case study shifts towards predicting the RUL of the water pump by leveraging real-time sensor data insights. The third case study delves into prognosticating critical operational conditions in the hydraulic rig, emphasising the importance of sensor-driven monitoring in identifying potential issues and impending failures. Together, these case studies show the value and the potential of Gaussian process in enhancing equipment reliability and minimising downtime in the mining industry.

To detail, case study 1 shows the effectiveness of the proposed composite kernel scheme in learning synchronously the multiple patterns existent in the data. The periodic kernel is a critical component as it is adept at modelling cyclically repetitive patterns. This capacity makes it ideal for identifying predictable operational fluctuations or periodic occurrences relevant to equipment operation. In parallel, the use of a linear kernel can recognise the global linear trend within the data. Furthermore, the inclusion of the Matérn 5/2 kernel introduces elevated flexibility into the model, allowing it to reach a balance between capturing the overall smooth trend and adapting to the varying amplitudes and irregularities present in the data. In contrast, the remaining two composite kernel designs lead to suboptimal predictive performance, especially with respect to RMSE and MAE , compared to using the Matérn 5/2 kernel. The highest $R^{2}$ and *R* from the proposed model indicate a strong correlation between the model predictions and the actual observations, which signifies the model's ability to accurately reproduce the underlying patterns in the data. This is further supported by the model extrapolation results depicted in Figure 3, where the increasing amplitudes, periodic fluctuations and weak linearity are forecasted. However, it should be acknowledged that the model is unable to well capture small fluctuations and noise components present in the data but provides primarily consistent smoothness in the predicted patterns, indicating that the model overlooks the varying nuances in the data. Yet, Gaussian process has shown its ability in the given task to discern the most significant patterns of machine degradation. As a decision-support tool, the method has the potential to proactively conduct the machine health monitoring task and inform the maintenance unit of the possible future degradation trends, thereby allowing efficient and cost-saving repair and replacement activities.

In case study 2, despite the data plots do not readily depict visible linear patterns, clusters and variations are evident. Hence, periodic kernel and linear kernel are not adopted. The RUL estimations of the two involved models shown in Figures 7 and 8 reveal that, despite some degrees of fluctuation, both models are able to capture the principal deterioration trend of the pump in the last 60 h of its function on the testing set. Notably, both models converge to estimate the remaining 20 h of operational time closely approaching zero, suggesting an early warning of failure occurrence. Decision-making-wise, this result implies a conservative maintenance strategy for a mining company. Following such a strategy, the organisation should conduct precautionary inspections and early preparation of spare parts, as well as deploy relevant maintenance resources, including workforce and budget allocation, for the pump that has not yet reached its projected end-of-life. These practices would enable the organisation to mitigate potential sudden disruptions to operations and expedite repair or replacement activities in the event of a breakdown, thereby safeguarding the reliability and continuity of the system's operation. Hence, despite the decline in model performance on the given evaluation metrics due to this premature estimation, this proactive stance still offers merits and remains a viable strategy in practice. Furthermore, a closer examination of the estimations reveals that the utilisation of the composition kernel yields more convincing estimations in the final 20 h. Its RUL estimation plot demonstrates a tighter fit to the actual RUL hours, indicating a higher level of precision in predicting the remaining lifespan of the pump. Additionally, while both models have captured the degradation trend for the initial 32 h out of 60, the composition kernel once again outperforms by exhibiting a better alignment between the predicted RUL and the actual RUL values in this period. On the other hand, both models fail to accurately estimate the RUL from the 33rd hour to the 40th hour where a sudden abruption in each estimation is exhibited. Indeed, it is naturally demanding to estimate the system RUL at each specific time stamp in great precision in the absence of a physics-based model (Zhang et al., 2016), which can define the degradation more precisely by considering mechanics, materials, environmental conditions, operational loads, etc. As such, RUL estimation by a data-driven model can only advise a rough deterioration trend acting as maintenance support. Yet, these mentioned deviations as well as overall existent oscillations in the estimations, while potentially stemming from the noisy data which poses challenges to the modelling process, still highlight the need for improving the models’ robustness to predict RUL values more linearly with fewer variations. This can include the refinement in the feature selection step and the exploration of more appropriate kernel function schemes together with hyperparameter search bounds. Overall, it can be found that the model utilising composite exponential and Matérn 3/2 kernels emerges as the preferred strategy for case study 2. It is true that the model with RBF kernel manages to capture the overall degradations by learning the non-linear relationships within the sensor records. However, the exponential component is more responsive to sudden shifts and irregularities, whereas the Matérn 3/2 component offers a balanced smoothness that can model gradual degradation trends while still being flexible enough to capture moderate variations. Thus, the composite kernel scheme has stronger flexibility in modelling the diverse range of data patterns and more accurate representation of long-term gradual changes and short-term irregularities as seen in the histograms and scatter plots, in contrast to using a single RBF kernel. As a result, it retains a sufficient correlation and a lower magnitude of the discrepancies between actual and predicted RUL values compared to its counterpart.

In case study 3, RatQuad kernel outperforms the remaining kernels on the validation set due to its leading $R^{2}$ . Indeed, compared with other kernels, RatQuad kernel has a stronger competency in recognising the non-stationarity and higher adaptability to abrupt transitions which are well exhibited in Figure 11. On the other hand, the third study opts to maximise $R^{2}$ in the kernel selection phase due to the nature of the underlying task. Given the limited number of distinct categorical values in each of the three rig conditions, the study objective becomes the identification and prediction of the conditional transitions between each state. Thus, prioritising the maximisation the coefficient of determination enables the model to focus on anticipating changes and anomalies in the machine’s behaviour, thereby leading to accurate forecasting of its condition evolution. Furthermore, case study 3 does not utilise any composite kernel, as a single kernel has been proven to be sufficient to learn the non-linear clustered patterns shown in Figure 11. Results from Table 9 indicate that the model achieves the best prediction performance on the rig's internal pump leakage condition. According to Figure 13, the model exhibits an ability to generate a temporal pattern that closely mirrors the actual variations, albeit with minor fluctuations. Observations from approximately the time units 1880–1950, 1970–2025 and 2120–50 show slight elevations in predicted conditions compared to the actual conditions. However, these deviations remain within an acceptable margin, and the predictions retain clear distinctions from other condition values. The same model obtains the second-best performance in the valve condition prediction task. Its declined *R* value still indicates a strong level of correlation between the prediction and the actuality. In fact, the transition of valve condition values holds the richest variation among the three target variables. Nevertheless, the forecasted temporal pattern illustrated in Figure 12 still has an overarching resemblance to the original pattern throughout the entire time series, with particularly decent proximity from the initial time point to around time unit 2030, and again from time unit 2070 to time unit 2120. The resemblance is preserved for the remaining time units, but the prediction shows a slight elevation to the ground-truth values. The decrease in $R^{2}$ and the increase in RMSE and MAE can be mainly attributed to the oscillations in the prediction, which leads to heightened deviations from the actual condition values. Finally, the prediction of the hydraulic accumulator variable results in lower RMSE and MAE compared to those of the internal pump leakage, but a further decrease in $R^{2}$ and in *R* is found. As illustrated in Figure 14, the forecasted temporal pattern is able to follow the general declination in the true data thus indicative of the rig's performance degradation. However, the model is hesitant in returning clear distinctions between each condition threshold, which suggests the further improvement in the model's differentiability for this condition. An important reason to the problem is due to hydraulic accumulator conditions having less frequent variations compared to the other two conditions. As such, the model's ability to capture useful patterns and its generalisability on new observations can be deteriorated.

A final remark would be related to uncertainty analysis and its importance in predictive maintenance management for a mining organisation. As depicted, the model in the first case study shows linearly increasing uncertainty estimates over time without sudden variations due to pattern-clear, low-noise sensor data. However, it starts to increase more evidently in the prognosis phase, where referenceable records are unavailable. This indicates the model becomes less confident in its results compared with the previous phases, and there is a higher risk that the forecasted patterns deviate from the actual machine degradation. Indeed, as the inferences are made beyond the range of observed data, the reliability of the Gaussian process model outcomes decreases. In the second case study, the uncertainty level alongside the RUL estimates is relatively low in the first 40 h, but it becomes consistently higher when the model provides its early warning of the terminal of the pump's life. In the third case study, the model has the strongest confidence in the prediction for the condition of the internal pump leakage followed by the valve condition, whereas the predicted outcome for the hydraulic accumulator is the least reliable due to its overall high uncertainty measurements. Furthermore, all three conditions follow a similar gradual reduction in uncertainty levels over the course of the testing data due to the model learning progressively the underlying pattern within the data from the temporal dynamics. The aforementioned suggests the strong necessity for cautious interpretation and implementation of the model estimations in predictive maintenance where the uncertainty remains innate. A thorough awareness and comprehension of the uncertainty measurements can help maintenance personnel assess the confidence level of the model predictions in practice. Correspondingly, the personnel can optimise their maintenance tasks strategically. For instance, in situations of high uncertainty, frequent inspections and precursory scheduling of part replacement activities should be planned. On the other hand, when uncertainty levels are low, the maintenance intensity can be adjusted to align with model's recommendations. This adaptive approach not only enhances machinery performance but also contributes to cost-effectiveness and operational efficiency. Furthermore, uncertainty analysis allows maintenance crews to allocate resources efficiently. For instance, if prediction uncertainties decrease for certain components within a mining system, mechanicians may focus resources on other areas with persistently high uncertainty to prepare for any unexpected breakdown. This targeted resource allocation ensures that maintenance efforts are directed where they are most demanded. Furthermore, from a broader perspective, a predictive maintenance programme should not solely rely on the model estimations but instead integrate them with domain knowledge, expert judgement and other available diagnostic information to formulate comprehensive maintenance strategies. A predictive maintenance model such as a Gaussian process-driven approach can provide a prognostic quantitative view of a machine's health status, but it should be regarded as a supportive tool rather than a conclusive verdict. The final maintenance decision should always conform to the actual operation progress, the real-time machinery condition and the financial circumstance of the company. That said, the organisation should equally consider practical needs and model outcomes. This way, stakeholders can adopt a more holistic and informed approach to maintenance management.

## Conclusions

Mining machinery needs adequate maintenance to ensure its reliability in rigorous mining operations. In comparison to the traditionally utilised maintenance programmes known as reactive maintenance and scheduled maintenance, applying predictive maintenance for the machinery permits a proactive identification of faults and anomalies before they escalate into costly failures, thereby minimising unplanned downtime and production losses. This paper showcases the Gaussian process for predictive maintenance strategy of mining machinery. Empowered by real-time sensor data analytics, Gaussian process models with different kernel function designs are developed and adopted in three case studies. Results from the first study indicate that the model is viable in deriving highly meaningful machine degradation trends for future reference. In the second study, where sensor data are noisy, the method provides an anticipatory maintenance policy by generating a reserved yet justifiable estimation for the lifespan of the water pump in its terminal phase of service. In the third study, the devised single Gaussian process model can generate three informative prognostications concurrently illustrating the transition and degradation patterns of three key conditions in the hydraulic rig. In each study, despite the model estimations offering valuable insights, they present varied degrees of discrepancies, which requires the maintenance unit to take judicious interpretations and actions within the context of broader operational considerations. Furthermore, each model generates uncertainty quantifications that show the model's confidence towards its corresponding outcome. The uncertainty underscores the importance of dynamic monitoring and adaptation in the discourse of maintenance activities. Together, integrating the estimations and uncertainties with qualitative and quantitative assessments in practice would enable a more robust and effective predictive maintenance strategy, ultimately enhancing the performance and longevity of the system.

In the current work, the kernel selection is a relatively subjective and cumbersome task since the optimal design for a case study is the result of iterative trials and comparisons among some pre-defined popular kernel configurations. To accelerate the determination process, enhance the objectivity and maximise kernel performance, a possible future work could be to integrate a Gaussian process-based predictive maintenance framework with an automated kernel selection module. Another improvement could be the development of a real-time diagnostic and prognostic maintenance tool. The model can receive and process up-to-date sensor data representing the operation dynamism in the mining machinery. The approach ensures that the model remains synchronised with the latest machine operational status. Thus, it can continuously calibrate predictive outcomes according to varying data patterns and machine conditions. This effort will optimise model responsiveness and accuracy in maintenance procedures, enabling timely and reliable prognostic interventions in a rapidly changing landscape in mining operations. Furthermore, the present work, which remains in the realm of predictive maintenance, could benefit from shifting to a prescriptive maintenance programme. Prescriptive maintenance involves hierarchical diagnosis and prognosis across multiple interconnected components and systems rather than focusing on individual elements. In addition, it can recommend specific actions to address potential issues identified through predictive analysis.
